# Genomic Variation, Evolvability, and the Paradox of Mental Illness

**DOI:** 10.3389/fpsyt.2020.593233

**Published:** 2021-01-21

**Authors:** Camillo Thomas Gualtieri

**Affiliations:** North Carolina Neuropsychiatry, PA, Chapel Hill, NC, United States

**Keywords:** autism, schizophrenia, genomic variability, evolvability, missing heritability, copy number variation, neural Darwinism

## Abstract

Twentieth-century genetics was hard put to explain the irregular behavior of neuropsychiatric disorders. Autism and schizophrenia defy a principle of natural selection; they are highly heritable but associated with low reproductive success. Nevertheless, they persist. The genetic origins of such conditions are confounded by the problem of variable expression, that is, when a given genetic aberration can lead to any one of several distinct disorders. Also, autism and schizophrenia occur on a spectrum of severity, from mild and subclinical cases to the overt and disabling. Such irregularities reflect the problem of missing heritability; although hundreds of genes may be associated with autism or schizophrenia, together they account for only a small proportion of cases. Techniques for higher resolution, genomewide analysis have begun to illuminate the irregular and unpredictable behavior of the human genome. Thus, the origins of neuropsychiatric disorders in particular and complex disease in general have been illuminated. The human genome is characterized by a high degree of structural and behavioral variability: DNA content variation, epistasis, stochasticity in gene expression, and epigenetic changes. These elements have grown more complex as evolution scaled the phylogenetic tree. They are especially pertinent to brain development and function. Genomic variability is a window on the origins of complex disease, neuropsychiatric disorders, and neurodevelopmental disorders in particular. Genomic variability, as it happens, is also the fuel of evolvability. The genomic events that presided over the evolution of the primate and hominid lineages are over-represented in patients with autism and schizophrenia, as well as intellectual disability and epilepsy. That the special qualities of the human genome that drove evolution might, in some way, contribute to neuropsychiatric disorders is a matter of no little interest.

## Evolvability and the Paradox of Mental Illness

There remains a gaping hole in Darwinian psychiatry's account of mental disorders: there are no good explanations of why human brains seem to malfunction so often, and why these malfunctions are both heritable and disastrous to survival and reproduction. That is, there is still no good answer for why such susceptibility alleles have persisted despite thousands of generations of natural selection for adaptive human behavior (1).

Nothing in biology makes sense except in the light of evolution (2) but, in light of evolution, mental illness does not make sense. Consider autism and schizophrenia. They begin early in life and are disabling during the reproductive years. In the language of natural selection, they compromise reproductive fitness. They are associated with decreased fertility, yet their prevalence is undiminished. The heritability of autism and schizophrenia is high (3–6), yet both are characterized by severe social impairment and are associated with low reproductive success.

The persistence of such disorders from one generation to the next is only one of several instances where mental illness is a challenge to Darwinian principles. For example, the problem of missing heritability (7). Autism and schizophrenia are thought to be the consequence of multiple genes of small effect; yet genome-wide association studies association studies have identified hundreds of such genes, and together they account for only a small proportion of cases (6, 8). The contribution of individual genetic variants and their cumulative action to mental disorders is disconcertingly small, usually <10% (9).

Another problem is variable expression, that is, the same genetic variant with various phenotypic expressions. The same genetic aberration may be associated with autism or schizophrenia or intellectual disability or epilepsy, or other neuropsychiatric disorders or combinations thereof (10). Variable expression is illustrated by the fact that mental disorders do not “breed true” but occur in different forms in family members (11–13).

Even within defined diagnostic boundaries, patients differ in virtually every salient characteristic, including symptoms, intellectual and functional abilities, neurocognitive strengths and weaknesses, neuropathological correlates, prognosis, and response to treatment. This observation has led to the idea of taxonomic “spectra,” e.g., the autism spectrum, the schizophrenia spectrum, the spectrum of mood disorders, etc. Implicit to the idea is that within every diagnostic category, mental disorders show a continuum of severity, from mild and subclinical cases to the overt and disabling. Most mental disorders occur in pure form in small numbers of individuals and in partial or subclinical forms in a great many more. One presumes a dosage effect, but a dose of what? (14–17).

New developments in genomic medicine have begun to illuminate the irregular behavior of complex diseases in general and mental disorders in particular. They are particularly salient to the neurodevelopmental disorders (NDD) and the focus here is on autism, a prototypical NDD, and schizophrenia which is increasingly recognized as such (18–21). NDD are conditions that originate during gametogenesis/embryogenesis and affect neural development (22). As a family, NDD are diverse in their clinical characteristics and prone to high rates of co-occurrence with other NDD. They are highly heritable but, in most instances, studies have only discovered multiple genes that are probabilistic in their association. Many if not most NDDs arise from structural changes to DNA; e.g., aneuploidy (Down syndrome), simple sequence repeats (Fragile X syndrome), and copy number variants (intellectual disability, epilepsy, autism and schizophrenia, and other mental disorders).

Structural variation, however, is only one chapter in an evolving story. It is just one of many irregularities that characterize the human genome, which we are learning to be uniquely dynamic and mutable. Techniques for higher resolution genomewide analysis highlight the irregular and unpredictable behavior of the genome, endowed as it is with a high degree of variability. It has served the hominid lineage for better and worse. Genomic variability accounts for no small proportion of the missing heritability of complex diseases (7, 23). It has also presided over the runaway evolution of our lineage over the past two million years, and especially the past hundred-thousand. The complex and adaptable human brain reflects a genome that is uniquely mutable and responsive to challenging environments.

The relevant principle is *evolvability*, a species trait that describes the capacity to generate heritable variations (24). The essence of evolvability is inter-generational and inter-individual variability. Phenotypic variation drives natural selection, but variation ultimately derives from the variability of individual genotypes; evolvability describes a genome that can generate a spectrum of phenotypes ranging from major evolutionary innovations to small changes between generations (25, 26). A dynamic, mutable genome is also unstable; it is responsive to life events, especially early ones, and vulnerable to insults of various kind throughout life (24, 27).

Perhaps mental disorders do not make sense in light of traditional genetics, but they make good sense in terms of evolvability. Variability is a highly evolved characteristic of the human genome. It rendered the hominid lineage especially *evolvable*, and humans uniquely adaptable. A dynamic genome is evolvable because it can generate a wide range of phenotypic variations in a comparatively short period of time. It is able to change not only at random but also in response to local exigencies. It has more tools in its kit than random point mutations; if mutational events that directly affected protein-coding sequences were the only available molecular mechanism to generate new variants, adaptive evolution would be ponderous and slow (25, 28). Primates would still be waiting in the trees for successive mutations to occur. Point mutations are insufficient for explaining the runaway evolution of the hominid brain. They can't explain why humans are so different from chimpanzees, why selection might favor genes for post-reproductive longevity, how signal human traits evolved as quickly as they have and how the extraordinary diversity of the human condition can arise from fewer than 25,000 protein-coding genes.

In the special case of hominid evolution, evolvability has been both an independent and a dependent variable. It is a general characteristic that promotes variation, but in our lineage it has been trait under positive selection (29). To support the trait of adaptability, hominids' special traits were agents of persistent and ongoing selective pressure. Social cooperation, abstract intelligence, language, speech and tool-making, and post-reproductive longevity rendered hominids uniquely adaptable. At the same time, they also generated selection pressures in favor of those very traits. Brain evolution, therefore, made hominids adaptable and also positively selected the trait of evolvability. The virtuous circle has been described as runaway evolution (30).

That the special qualities of the human genome that drove our evolution might, in some way, contribute to complex diseases, NDDs and mental disorders is a matter of no little interest.

### Origin Stories

The problem of High Heritability and Low Reproductive Success (HHLRS) is paradigmatic. It has been addressed many times and from different perspectives, especially with respect to schizophrenia, a condition that occurs with the same frequency in all the nations and, as far as we know, always has. The quality of our understanding has steadily progressed, beginning with explanations that posited adaptation, thence to those that emphasize adaptability; beginning with evolution as an origin story and later concerned with evolvability as the consequence of a mutable and dynamic genome.

“Adaptationist” theories suggest that a trait embedded in the schizophrenic genome generates phenotypic variations that are advantageous to individuals who do not express the full phenotype. Perhaps the unique characteristics of psychotic individuals were more valued in ancestral times. Psychotic individuals may have been charismatic leaders or shamans (31). Their lower threshold for threat perception may have been useful in times when life was nasty and brutish (32)—*Just because you're paranoid doesn't mean they aren't after you*^1^. The relatives of schizophrenic individuals may be more resistant to disease (33, 34). An idea still current is that mental disorders are associated with creativity (35)—*The romantic view is that illness exacerbates consciousness. Once that illness was TB; now it is insanity*^2^. It was supported by a recent Swedish study that reported individuals with bipolar disorder and the healthy siblings of people with schizophrenia were overrepresented in the creative professions (36) and an Icelandic study that found the same association based on polygenic risk scores (37).

In a similar vein, the occurrence of autistic styles of thinking in the first degree relatives of autistic individuals suggests traits like hyper-systematizing, preference for visuo-spatial relationships and detail-focused processing are adaptive, not only for individuals, but as we learned from the accomplishments of scientists and mathematicians with autistic traits, for the distributed intelligence of society. If autism were a single-gene disorder, it would suggest heterozygote advantage. If autism were a polygenic disorder, a Gaussian model might be relevant, with traits for autistic thinking normally distributed in the population, at the tail end of the curve “hyper-systematizers” would cluster, and beyond a certain threshold reside autism (38, 39).

Adaptationist theories propose that susceptibility alleles are maintained by antagonistic pleiotropy or balancing selection. In that case, probands and/or non-affected siblings would have higher fitness than the general population. In fact, individuals with autism and schizophrenia have lower fertility rates, and their siblings' are the same or lower than the general population (1, 40–44).

### Faustian Bargains

Many theories share the metaphor of a Faustian bargain; the human brain is complex, and complex systems are “intrinsically and irreducibly hazardous” (45). For example:

- As specialized functions evolved, biological “trial and error” produced individuals with advanced abilities and others with abnormalities, including schizophrenia (46).
- In the course of evolution, positive selection for cerebral flexibility allowed language to emerge, but the “by-product” was variation in psychological functioning, personality disorders and schizophrenia (47).
- The human brain, with its complex and recently evolved circuitry for social cognition, matured over a long span of time, a span that rendered it susceptible to genetic insults. “This susceptibility was the trade-off for the advantages gained in social cognition” (48).
- The same key genes that have been major contributors to the rapid evolutionary expansion of the human brain and its exceptional cognitive capacity also, in different combinations, are significant contributors to autism and schizophrenia (49, 50).

“By-products” and “trade-offs” refer to antagonistic pleiotropy, when alleles increase the evolutionary fitness payoff of one trait while simultaneously reducing it for another. Such theories posit that a species' predisposition to mental disorder has a selection advantage, albeit indirect. However, interesting, they lack empirical support.

More recently, theories of the HHLRS problem have implicated genomic variability. A high mutation rate characterizes primates in general and humans in particular; multiple, independent *de novo* mutations in many different vulnerable genes and genomic regions (25, 51–60). A theory based on polygenetic mutation-selection balance is that complex brain functions are the work of multiple genes; the genome already harbors a large number of mutations, and new mutations are occurring all the time. Every generation, therefore, carries the burden of old and new mutations, some favorable and some not. Because the distribution of mutations is assumed to be continuous in the population, the expression of positive and negative phenotypes is also continuous. The distribution of negative traits reflects the continuous nature of most (if not all) mental disorders. The “cliff-edge” model captures the non-linear amplification of negative traits that leads to severe and disabling mental illness (61).

Theories derived from a high mutation rate, unlike the others mentioned above, do not propose evolutionary trade-offs; i.e., the cost of complexity is met by the occurrence of neurodevelopmental disorders. The theory of polygenetic mutation-selection balance explains how fitness-reducing genetic variation is maintained in the population; harmful mutations are removed from the gene pool at a rate proportional to their effect on fitness, but “novel mutations occur all the time” (1, 53, 62–65). Thus, the occurrence of mental disorders is inevitable, fueled by deleterious mutations, rare at individual loci but ubiquitous in genomes (1, 66–68).

Addressing the HHLRS problem from the perspective of evolvability is not quite so pessimistic. Novel mutations do, in fact, occur all the time, including rare alleles with large effect, genomic transformations like copy number variants, mutations in non-coding regions of the genome and epigenetic changes in gene expression. They may persist for only a few generations but are continually replenished by virtue of high mutation rates (69). Mutations, however, are not necessarily random, but appear to occur in the human genome in accord with principles that are only now coming into focus. The complex, evolvable genome is prone to devastating errors but they occur in particular, knowable ways that open possibilities for anticipation and prevention.

### Genomic Variability and the Problem of Missing Heritability

Although single-nucleotide polymorphisms (SNPs) are the most abundant form of DNA variation in the human genome (67), new technologies have shown that individual variation is also the consequence of structural variants involving larger segments of DNA (8, 70–72). Two randomly selected human genomes differ by 0.1% when only SNPs are measured, but when structural variants are also measured, they differ by at least 1% (73, 74).

Structural variants may be rare, compared to 37 million or so SNPs but their large size increases the potential to affect gene expression (4, 75). Genes containing regulatory regions, exons and introns occupy about 5% of the genome and protein-coding exons only about 1% (76), while structural variants comprise no <55% of DNA (77) and perhaps as much as two-thirds (78). Thirty per cent of the human genome are microsatellites (79) and about a third are copy number variants (80–82). Structural variation is more common in humans than other mammals and occurs several times more frequently in neurons than other cells (54–58). Evidence from a wide range of common diseases indicates that genetic heterogeneity is a key characteristic of the human genome and that “most genetic control is due to rare variants” (4). The challenges that genetic analysis poses to mental disorders are well in accord with our new and developing appreciation of genomic behavior. Genomic variability is not only the fuel of evolvability but a window to understanding the origins of complex disease, mental disorders and NDDs.

## The Nature of Genomic Variability

### 1. Structural Variation

The hominid genome is not a static blueprint but a source of “gene nurseries” that play a role in gene innovation and adaptability (83). It is something that happened during the later course of evolution. Pre-primate evolution was probably driven by point mutations or whole-genome duplications (59, 84, 85). Ascending the phylogenetic ladder, mutations by genetic rearrangement have been progressively more important. Almost all of the genetic differences between humans and other primates are a result of duplications, deletions, inversions, insertions, and transformations (86, 87). For example, about 35 million nucleotide substitutions distinguish humans from chimpanzees. About half are transposable element insertions (88).

The high-resolution molecular scanning tools developed during this century have revealed a genome that is no less than restless. For example, aneuploidy traditionally referred to supernumerary copies of whole chromosomes (e.g., trisomy 21, Down syndrome). In recent years, novel aneuploidy syndromes have been identified, and the definition has been extended to include deletions, insertions and duplications of subchromosomal regions (89). A new term that includes classical aneuploidy and other structural variants is DNA content variation (DCV). Such variants are dynamic, fluid and unstable, both through germlines and in somatic events and they are ubiquitous in the human genome (74).

Structural variation is most likely to occur in non-coding regions of the DNA molecule. Non-coding regions are comparatively unstable and generate mutations and rearrangements at a high rate (90–92). They are especially prone to genomic rearrangements. As it happens, the human genome contains more non-coding DNA than any other animal or plant. Among microorganisms, <25% of DNA is non-coding. In plants and lower animals, about 60% is non-coding; in primates, the proportion is higher. In humans, it is 98.5% (93).

The human genome is particularly enriched in both number and length of retrotransposons (94). The propagation of *Alu* elements is coincidental with the fast evolution of segmental duplications in the primate genome, which grew as a result of a major burst in *Alu* activity 25–55 MYA; ours have continued to expand (56, 59). Compared to chimpanzees, humans have nearly three times as many *Alu* elements (88).

Copy number variants (CNVs) are duplications or deletions that are >1,000 base pairs in length. At least half of the CNVs thus far detected include protein-coding regions and affect the behavior of the relevant genes. The functional impact of CNVs extends across the full range of biology, from gene expression to cellular phenotypes (70, 95) and to all classes of human disease with an underlying genetic basis, whether inherited or sporadic (74, 96). They are especially relevant to autism and schizophrenia.

#### Aneuploidy Is Common in Brain

The human brain is a genetic mosaic. In the adult cerebral cortex aneuploidy, broadly defined, is estimated to occur in no fewer than 30–50% of neurons, many times more frequently than in somatic cells (97). Fetal neurons develop over a longer span of time than those of most other animals, and undergo more cell-divisions along the way. A prolonged span of mitotic activity, one presumes, makes such rearrangements more likely (98).

How the brain accommodates events that are potentially deleterious is a mystery. In spite of cellular variability and diversity and the consequent differences in gene expression, the functionality of the CNS is usually not compromised (99, 100). Aneuploid cells are capable of surviving the massive cell death that accompanies neurogenesis (101). They can differentiate into neuronal lineages and are integrated into active neural circuitry with the potential to influence normal brain functions (102, 103). DCV shows regional variation within the human brain, more in the frontal cortex and less in the cerebellum. Neuronal DCV concentration also differs among individuals (104). Genetically mosaic neural circuitries are part of the normal brain organization (105, 106) and reflect the structural and functional mosaicism of brain itself, its neuronal diversity and expansive range of behavior (97).

The prevalence of neuronal aneuploidy captures two principles that we shall rely on. One is that *brain is an evolutionary system*. From this perspective, maintenance of neutral or beneficial aneuploidies is the end-result of selective pressures (107). Extreme forms of aneuploidy are eliminated during fetal development by programmed cell death, while other neurons survive and contribute to neural organization. One assumes that the loss or gain of genetic material may render some cells more “fit” than others, perhaps by increasing stress resistance or enhancing functional capacity (97).

The second principle is that *high mutation rates confer adaptability*. Genomic diversity prepares developing brain for the multitude of tasks before it. A more diverse neuronal population is better-equipped to adapt to challenging environments; a lineage thus endowed is under positive selection for genomic variability and evolvability.

Although mature neurons are terminally differentiated cells, they remain capable of generating structural variants. When they are stressed, neurons seem to be able to reactivate elements of the replication mechanism (103). Such “cell-cycle events” may be pathological (108); they have been observed in several human neurodegenerative diseases, including Alzheimer's disease and ataxia telangiectasia. Alternatively, they may be an attempt at neuronal self-protection. The ectopic expression of cell cycle markers may be “a desperate attempt of a neuron under stress to protect itself” (109). It is not a paradox but another principle of evolvability: *mechanisms to increase robustness are also, in some circumstances, fragile, and vulnerable to pathology*.

That genetic plasticity of post-mitotic neurons is mostly adaptive is captured by the frequency with which mobile elements can change their position within the genome, either by a DNA-based (transposition) or an RNA-based (retrotransposition) mechanism. The latter is of particular interest because it is a form of plasticity that responds to early life experiences (110, 111).

#### Structural Variation in Autism and Schizophrenia

DCV contributes to phenotypic diversity, adaptability and individual differences in brain organization. It also plays a role in disease, albeit one that is quite variable (89, 97). A number of recent studies have provided compelling evidence that autistic, schizophrenic, and bipolar patients are more likely to possess CNVs in their genome, especially deletions of genomic regions (112, 113); no one CNV in particular, however, but rather any of a number of rare CNVs (11, 72, 114–120).

Compared to healthy controls, individuals with schizophrenia are three times more likely to harbor rare structural mutations. The risk is even higher in subjects with early onset schizophrenia. Each rare mutation disrupts a different gene or genes and the disrupted genes are disproportionally involved with signaling and neurodevelopment (4, 113). The burden of CNVs in schizophrenic patients ranges is low, from 2.4 to 10% (121, 122). However, CNV analyses with better genome coverage will probably discover many more relevant associations (123). When microdeletions and microduplications >100 kb were identified by microarray comparative genomic hybridization and validated by high-resolution platforms, novel deletions and duplications were found in 15% of individuals with schizophrenia and 20% of young-onset cases (vs. 5% in controls) (124).

The prevalence of structural variants in autistic patients, on the basis of less sensitive genomic scans, is usually given as 5–10% but may be as high as 28% (112, 114, 120, 122, 125). Although the vast majority of CNVs are inherited, CNVs that occur as *de novo* mutations are more commonly associated with autism (and schizophrenia). *De novo* CNVs are found with higher frequency among sporadic cases, whereas inherited CNVs are more common in familial cases (121, 126–129). With better technology, the prevalence of structural variants will likely be higher (95, 128, 130, 131).

It probably will be but what will it mean? Structural variants, interesting as they are, may simply join the long list of “multiple genes of small effect” and the even longer list of epigenetic and non-genetic factors associated with autism and schizophrenia, with no one making more than a small contribution (132). Many CNVs are functionally neutral; healthy individuals carry, on average, about 11 CNVs (57). Even the CNVs that are known to be associated with a neuropsychiatric disorder are present in low concentrations in healthy controls (49, 128, 133–138). CNVs are not, as a rule, highly penetrant (139–142). Further, when CNVs are expressed, the phenotypes are variable: schizophrenia or autism, but more often developmental disabilities, congenital malformations, or other mental disorders (11, 125, 140).

### Genomic Variability: 2. Variable Expression

*Reduced penetrance* refers to individuals with a specific genotype, but the clinical phenotype is not expressed or is expressed in a lesser form. *Variable expression* is the degree of variation in a clinical phenotype in individuals who carry a specific genotype (143). CNVs manifest both.

CNVs known to be associated with autism or schizophrenia are also found to occur in association with other psychiatric or neurodevelopmental conditions. A good example is the 22q11.2 deletion. The deletion occurs in only about 1% of schizophrenic patients, yet it is the strongest DNA-based risk factor for schizophrenia identified so far. Individuals with the 22q11.2 deletion have a 20-fold increase in risk for schizophrenia. Nevertheless, individuals with the deletion may be perfectly normal; or they may have developmental delay, congenital malformations (144, 145), generalized epilepsy (146, 147), intellectual disability (148), learning disability, autism, ADHD, anxiety, depression, OCD, or bipolar disorder (11, 97, 113, 118, 149–155). Variable expression is certainly the case for many other deletions and duplications [e.g., 1q21, 15q13.3, and 16p11.2; (113)]. Even when a structural variant and the alleles therein are well-defined, or when a repeat number is correctly counted, the clinical consequences are unpredictable. In a kindred harboring a translocation disrupting *DISC1*, carriers had schizophrenia, bipolar disorder, major depressive disorder, cognitive impairment or no mental disorders at all (116, 125). It is perplexing but typical not only of complex diseases but complex traits in general (115).

### Genomic Variability: 3. Epistasis

Most phenotypes result from intricate gene interactions. These interactions, recognized as deviations from additive genetic effects on the phenotype, and collectively called epistasis (156). It is a universal characteristic of complex genetic traits. It is one more aspect of genomic behavior that accounts for the phenotypic variations that occur with mutations in the same gene (157). A mutation may be benign or beneficial to one individual but deleterious to another individual; it is contingent on an individual's “genetic background” (158). “Sign epistasis” means that a mutation is beneficial on some genetic backgrounds but deleterious on others (159).

“Epistasis” was coined to describe the suppression of an allelic phenotype by an allele at another locus (160). It is more complicated than gene B influencing the expression of gene A; “higher order” epistasis involves interactions among multiple mutations (161, 162). Antagonistic epistasis among deleterious mutations and synergistic epistasis among beneficial mutations represent positive epistasis, whereas the opposite situations represent negative epistasis. Intra-gene epistasis results from effects of mutations on RNA stability and enzyme activity and inter-gene epistasis may result from protein interactions and the structure of metabolic networks (156, 163).

To say that a disorder is the consequence of “multiple genes of small effect” understates the magnitude of the problem. The results of multiple genes acting together may be additive or multiplicative, linear or non-linear. Epistasis is also affected by events in the environment of the cell (164, 165) and the developmental stage at which they occur (166). Within a gene, different alleles can interact epistatically with different gene sets (167).

Divergent phenotypes emanating from identical genetic variation(s) are consequences not only of epistasis but also (in varying degrees and combinations) pleiotropy and locus heterogeneity, environmental factors, epigenetic mechanisms, stochastic events, dose and timing of gene expression, and RNA regulatory elements (4, 118, 138, 168–172). Some variants may only affect risk if they co-occur with other genetic or environmental risk factors (115).

The Darwinian “problems” we cited earlier were the heterogeneity of disorders even within well-defined categories, their occurrence in pure form in small numbers of individuals and in partial or subclinical forms in a great many more, the fact that most such conditions do not “breed true” but are present in different forms in family members, comorbidity, and the HHLRS problem (11–17). The question is not why they happen but, knowing what we now know, how could they happen in any other way.

### Genomic Variability: 4. Gene Regulation

The main effects of DNA structural variants are commonly attributed to changes in gene expression and its regulation. The latter, however, represent an entirely different dimension of genomic variability. Gene expression is the work of multiple genes and other DNA segments, proteins, and RNAs of varied stripe, as well as epigenetic changes to DNA and chromatin. Together, they comprise the genome's control architecture and participate in countless gene regulatory networks (GRNs). Networks of regulatory genes occupy more DNA than protein-coding genes do (173, 174).

Variation in gene expression levels is abundant within and among populations; quantitative differences in gene expression are responsible for a significant amount of the variation represented by individual differences (89, 175). Gene regulation is itself a heritable trait. When variation in gene expression phenotypes is compared among unrelated individuals, among siblings within families and between monozygotic twins, there is a strong genetic contribution to variation in the level of gene expression (176, 177).

Gene expression microarrays and transcriptome sequencing have revealed remarkable natural variation in gene expression levels within populations as well as between species (178). Regulatory variation within and between species is thought to explain a large proportion of phenotypic diversity of life. It is believed that most complex traits originate in non-coding regions that affect gene regulation (179).

- *GRNs are the basis of organization and stability*. They are models of dynamic complexity with modular structures that were present at the base of the metazoan tree (93). Their stability is a function of modularity, feedback loops and the redundancy of the genetic material upon which they operate. They are able to withstand gene disruptions due to mutation or environmental stress (93, 180).
- *GRNs are organized hierarchically*. GRNs are organized hierarchically and control the nature of available variation by “packaging” genetic modules for selection (181). Thus, they are agents not only of stability but also evolvability.
- *GRNs also reflect the fact that the organism is a complex adaptive system*. In GRNs, information from the cell state and the outside environment is translated into the correctly timed expression of the appropriate gene products (182). GRNs have been called the “nexus of physiological adaptation” (183).
- *GRNs are a source of endless variation*. For most genes, transcript structure and expression level are not only highly variable but functionally independent (175).

### Genomic Variability: 5. Epigenetics

Epigenetic changes participate in GRNs by influencing whether a gene is expressed, when it is and to what degree. Histone (or chromatin) modification changes the structure of a core octamer that contains two copies of the histones H2A, H2B, H3, and H4 (humans have them and chimpanzees don't). Histone remodeling ensures that DNA remains accessible to the transcriptional machinery (184–187).

DNA methylation is another epigenetic mechanism that can affect gene expression by making cytosine binding sites more or less accessible to transcription factors; or it may attract proteins that are themselves gene repressors. Variable DNA methylation is the loss or gain of methylation at CpG dinucleotides. Variable DNA methylation is the loss or gain of CpG dinucleotides; levels of methylation vary among individuals and change at different stages of life. Patterns of methylation or hypomethylation at different sites appear to be associated with longevity or, alternatively, aging-related diseases like cancer and Alzheimer's (188–191).

Non-coding RNAs (ncRNA) are a third epigenetic mechanism. ncRNAs are generated by intergenic or antisense transcription, usually at introns, and comprise more than 90% of transcriptional output from the genome. ncRNAs remain tethered to their transcription site, serving as allelic markers and lending spatial and temporal specificity to gene expression (173, 192). An example of temporal specificity are the 35 ncRNAs that govern the development of dopaminergic neurons from neural stem cells. They are differentially expressed between progenitor and mature states and, in all probability at different stages of the life cycle (193).

Although epigenetic mechanisms are most active during embryogenesis and early life, they remain active throughout life, meaning that life experiences can make enduring changes in the genome. Further, epigenetic changes can be passed on. Phenotypic variations unrelated to variations in DNA base sequences may be transmitted to subsequent generations of cells or organisms; an epigenetic trait is “a stably heritable phenotype” (194–196). Epigenetic inheritance is not quite the same as the Lamarckian theory of inheritance of acquired characteristics, but it's not all that different. It allows genetic variants that do not change the *mean* phenotype change the *variability* of phenotype. However, epigenetic modifications can also affect the probability that a region of the genome will mutate [e.g., single-base and transposon-mediated mutations and translocation; (197)]. Therefore, not only may epigenetic modification promote heritable phenotypic variation, but can also facilitate genetic evolution by modulating mutation rates across the genome (198, 199). Epigenetic variation, therefore, is a powerful mechanism for evolutionary adaptation to changing environments.

Epigenetic participation in GRNs is illustrated by the remarkable diversity of genetically identical cells and organisms even when they have identical environmental exposures. Cloned animals, for example, may have different phenotypes at birth. In monozygotic human twins, gene expression is four times more dissimilar in older subjects (50-year-old monozygotic twins) in comparison to younger subjects (3-year-old monozygotic twins). Autism and schizophrenia both show surprisingly high frequencies of phenotypic discordance in monozygotic twins. In one study, genomic DNA extracted from leukocytes of male MZ twins discordant for schizophrenia was found to have significant differences between the twins at sites that are closely associated with CpG islands and gene expression (200, 201). Similar differences in methylation patterns have been noted in MZ twins discordant for autism (202).

Epigenetics reflects three principles long familiar to psychiatrists:

1. Experience influences development and the effects may be long-lasting.
2. The maternal contribution to development is important on many levels, including the transmission of gene expression patterns by epigenetic programing *in utero* and early life.
3. Epigenetic programming is particularly robust in early life but epigenetic re-programming, modified by experience, occurs throughout the lifespan.

### Genomic Variability: 6. Stochasticity, Noise

Another principle that psychiatrists encounter, perhaps more often than they like, is captured by the stochastic behavior of gene regulatory networks. The generation of gene products is necessarily sensitive to unpredictable fluctuations. Gene regulation is intrinsically “noisy” (203).

Transcription factors are proteins expressed by genes which, in turn, control the expression of genes. Their dynamics are constrained by a highly structured, densely tangled intracellular environment where DNA, RNA, and proteins may be present and active with only a few copies per cell (181). Since typically 30–100 regulatory proteins per gene are used as transcription factors (204), a corresponding number of genes must go through their individual cycles of expression in a perfectly synchronized manner; otherwise, a shortage of a few transcription factors may lead to drop-out from the regulatory process and a halting of big sections of transcription machinery (205). When a transcription factor initiates gene expression, its effects are amplified by cycles of epigenetic reprogramming; normal cell activity involves thousands of transcription factors operating in parallel with epigenetic mechanisms. Thus, there are ample opportunities for “noise” or stochasticity—to arise. A small number of epigenetic changes, or a single one, may have a net effect on multiple downstream targets. Environmental signals can affect the activity of transcription factors and epigenetic complexes to regulate gene expression (93, 192).

The modular structure of GRNs is a stabilizing factor but there is no overlying regulatory system, in the sense that “regulation” is used in systems control theory (181). If anything, GRNs are self-organized, responding to differences in the internal states of cells; to the effects of subtle environmental differences, such as morphogen gradients during development; to predictable processes such as cell cycle progression; to random processes such as partitioning of mitochondria during cell division; and to ongoing genetic mutations (206). They also manifest the inherent stochasticity of biochemical processes that are dependent on infrequent molecular events involving small numbers of molecules. Like people, they encounter innumerable opportunities to do something unpredictable.

Intellectual disability, autism and schizophrenia have been related to GRN's that regulate neurogenesis, neuronal connectivity, cell signaling, axon guidance, presynaptic pathways, post-synaptic protein complexes, cytoskeleton dynamics, intracellular signal transduction pathways, transcription regulation, and epigenetic modulation of the chromatin structure (207–211). Clinical studies of gene networks, however, are theoretical, relying on statistical associations among genes that are expressed in particular areas or that have known functions. Mapping the “interactome” directly has only been done in model organisms such as yeast, fruit flies, and roundworms.

### The Origins of Genomic Variability

It may be reassuring to clinicians to learn that the genetics of complex disease are no less complex than the phenotypes they generate but may wonder how it came to be that way. Genomic variability, in all its dimensions, has increased as evolution scaled the phylogenetic tree; a high mutation rate characterizes primates in general and humans in particular. It is telling that, of all the great apes, humans have less genetic diversity defined by the number of SNPs, but much more in terms of structural variants (212).

DNA content variation is more common in humans than other mammals; because it occurs several times more frequently in neurons than other cells we may assume it favors the evolution of a complex and adaptable brain (25, 54–60). Most of the structural rearrangements in the mammalian lineage are believed to have occurred in brain-specific genes (55). The duplication rate accelerated at the time of the common hominoid ancestor (57) and our own species is remarkable for numerous large segmental duplications (25, 60).

Structural rearrangements allow multiple forms of a gene to co-evolve and to rapidly reorganize the genome. Variation in the amounts and types of repetitive DNA varies between organisms and reflects how rapidly a species is capable of evolving in response to changes in its environment (213). It is believed to foster inter-individual genetic variability and variation from one generation to the next (168, 214, 215). The creation of novel genes by genomic transformation is said to have been “the major driving force in hominid evolution” (91) and “the *sine qua non* for evolvability” (24).

Changes in the gene regulatory machinery are another creative force in morphological evolution (55, 216). The proliferation of new regulatory genes coincided with the emergence of increasing organismal complexity, and they enabled organisms to develop new functionalities. Differences in gene expression are probably the real divisor between humans and chimpanzees; our genes are largely the same but the difference is accelerated gene expression changes in the human brain (58). During hominid evolution, mechanisms of gene expression have been elaborated to an extraordinary degree and this is especially true of genes that govern brain development (28, 58, 217, 218). The adaptability of the genome is further enhanced by epigenetic mechanisms and, as it happens, humans are the most epigenetically complex species (219, 220).

The convergence of genomic variability, morphologic evolution and the evolution of a complex, highly adaptable brain is contained within the concept of evolvability. Animals possess two systems with which to address the challenges of their environment: a neural system, that directs behavior and a genomic system that provides the wherewithal for behavior to occur. Both systems are composed of networks that are at once inordinately complex and also flexible. Their complexity and flexibility increase as one ascends the phylogenetic ladder. We humans are the carriers of genomic and neural systems that make us uniquely adaptable and, one may say, evolvable.

We also know that genomic events that conferred evolvability to the primate/hominid lineage are over-represented in patients with autism and schizophrenia, as well as intellectual disability and epilepsy. The irregular behavior of the genome is probably reflected in the evolution and organization of neural connectivity; it is certainly reflected in the irregular behavior of complex diseases in general, autism and schizophrenia in particular. The pictures are gradually coming into focus: the mechanisms of genomic variation, the vicissitudes of gene expression and their variable phenotypic consequences. As the particulars accrue, so do underlying principles. Genomic variability is clearly related to evolvability and to NDDs, but are the latter two connected? The question has been answered: “The same genes that were responsible for the evolution of the human brain are also a significant cause of autism and schizophrenia” (50). There are hazards, however, to facile answers. The issue isn't “genes” at all but the principles that govern their behavior. Three principles relevant to evolvability are reiterated in a clinical arena where the idea of evolvability is current, in studies of cancer.

### Evolvability in Real Life

*Cancer as an evolutionary system* is neither metaphor nor theory but an observation that has implications for diagnosis and treatment (221). Cancers are dynamic entities that never cease to evolve. They don't only grow; they “evolve according to well-understood principles of somatic selection, along trajectories that can be described by established methods for tracing phylogenies” (222). Individual cancer cells are reproductive units within a population and compete not only with non-neoplastic cells but also with other cancer cells that possess different genotypes. Neoplastic cells, especially solid cancers, generate additional mutations with new phenotypes, such as the ability to invade adjacent tissues, recruit blood supply, overcome nutritional deficiencies and resist immune attack (223). Their mutational patterns evolve stochastically and are highly diverse. Cancers contain, on average, 50 non-silent mutations in the coding regions of different genes; only a small fraction of the mutations are common to all tumors in a given class (224–226).

In cancers, genetic heterogeneity is “the fuel that drives selection” (223, 227). Cancer cells are notorious for their genomic instability. They show genomic rearrangements at the microscopic and submicroscopic level; mutations in coding regions, genomic loss or amplification, transpositions and transformations, aberrant methylation and expression profiles. Such mutations render cancer cells evolvable and, all too often, difficult to treat^3^ (228, 229).

The evolvability of cancer cells captures three relevant principles. The first is that *high mutation rates confer adaptability*. Cancer cells are subject to stresses—hypoxia, nutrient depletion, immune surveillance, chemotherapy, —and they show that high mutation rates occur when cells are stressed. Mutations accelerate adaptation and cells with the requisite phenotypes are selected (230–232). This observation has wider import; it belies the assumption that mutagenesis is random, constant, and gradual. Mutations occur more frequently when cells are maladapted to their environments, and the mechanisms that drive mutation tend to target specific genomic structures (233).

The second principle is that the genome is balanced between two *evolutionary traits, stability, and variability*. Stability, or robustness, is the ability to remain adapted to existing conditions in spite of perturbations. Cells that are genetically unstable—for example, those with certain CNVs—are predisposed to neoplastic transformation (229, 234). Cancer cells are unusually robust because high mutation rates render them more variable, more likely to advance in the face of hostile environments (235).

The third is that *the human genome has achieved a good balance*. The hominid lineage has evolved successfully while maintaining an array of DNA monitoring and repair enzymes that render most mutations an evolutionary dead-end. That is why cancers, like severe, disabling mental disorders, are comparatively rare events. Our genome derives disproportionally from individuals who had effective mechanisms for suppressing the conditions (223, 236). Nevertheless, mutations occur and continue to exercise effects, not always good ones. The balance is not perfect. Cancers, like mental disorders, continue to occur. Mechanisms for mutation prevention and suppression are imperfect. The accumulation of new genetic variants may fuel evolution of the species but not all variants are beneficial to individuals.

### Neural Darwinism

Neoplastic cells compete not only with their host but with each other. The evolutionary expansion of neoplastic cells is a far cry from what happens among post-mitotic neurons in autism and schizophrenia, but the point is that evolution is more than an historical event; it is the product of biological processes and principles that remain active. So, if brain were regarded as an evolutionary system, the evolvable units would not be neurons but the connections that form among them—at a fundamental level, synapses and at the next, neural networks. If autism and schizophrenia are, in fact, disruptions of neural circuitry (237, 238), they may be conceptualized as aberrations in one or more evolutionary processes.

The development and maturation of brain is characterized by, among other things, the evolution of discrete interconnected groups of active neurons–-“cell assemblies” or neural networks (239). “Synaptic Darwinism” refers to synapses “replicating” by strengthening their connections and “mutating” by connecting new cells. “Neural Darwinism” is more than a metaphor. During the course of development and throughout life, there is always a vast array of potential connections to be made, but only the fittest survive (240). The axonal arbors of neurons tend to be in close proximity; competition results in strengthening the connection with one neuron and withdrawal of the rest. Onle certain connection patterns are *selected* in order to form optimal configurations (241); alternatively, one may say that neurons *compete* to form effective connections (242) “What could be analogous in the case of synapses to genetic mutations? We believe the obvious analog to genetic mutation is structural synaptic change” (243).

“Competition” among neurons is reflected at the level of molecules; in living cells different molecular species compete for binding to the same molecular target. A relevant example is the competition of genes for the transcription machinery or the competition of mRNAs for the ribosome. In transcription and translation networks, competition takes place within large-scale networks that typically have hundreds to thousands of competitors, all at a relatively low concentration. Many different genes compete for RNA polymerase and transcription factors, microRNAs compete for mRNA and thousands of different transcripts compete for a common set of ribosomes and translation factors. Translated proteins compete for a common folding and transport machinery and when they are to be removed they compete for a common degradation machinery (244–246).

Competition among alternative pathways occurs in the behavior of neural networks and the mechanisms of cognitive control (247, 248). In the lateral amygdala, neurons with increased excitability during training outcompete their neighbors for allocation to an engram (249). Representation in the visual system is competitive; both top-down and bottom-up bias influences the ongoing competition (250). Awareness itself is a bottleneck through which only a small amount of neural activity is successful in coming to one's attention (251, 252).

The evolvable human genome gives rise to neural systems that are themselves evolvable. The principle that mutations confer adaptability is equally pertinent to the genome and the neural connectome; variability in the former is a homolog of plasticity in the latter. So also does the principle of balance. The neural connectome depends on the expression of many genes involved in neural activity, including the behavior of receptors, membrane transporters, enzymes for neurotransmitter synthesis or degradation, cytoskeletal and vesicular proteins, signaling and effector proteins, and regulators of transcription and translation (253). The variable expression of so many genes lends bias to the development of neural connectivity. Autism and schizophrenia originate in networks of DNA, nucleotides and proteins that preside over neurogenesis and neural migration, synaptogenesis, and arborization. One can say that autism and schizophrenia are “disruptions of neural circuitry,” or one might better say that certain genes lend bias to the evolution neural circuity. “Disruption” conveys either/or; “bias” is consistent with the clinical heterogeneity of those disorders. The competition at every level to form optimal connectivity is biased against a perfect balance.

### Robustness Is the Balance Between Stability and Variability

Complex adaptive systems, such as the genome and the neural connectome, are networks comprised of a hierarchy of networks. Coherent behavior in such systems arises from the interplay among many individual agents (254, 255). Networks and the agents within them are subject to events in the environment and to inevitable stochastic variations, and to all are available alternative, competitive pathways. A system is robust if it optimizes the balance between cooperation and competition, stability and variability, randomness, and regularity; thus, a species is robust if it can maintain essential functions yet maintains the potential to evolve (256, 257). The optimal balance is a robust system that is stable but also flexible and adaptable. Thus, the genome responds to stresses with adaptive mutations and the neural connectome responds with adaptive plasticity. How well cells, organisms, and species achieve that balance in the face of innumerable unforeseen events is the essence of evolvability.

The capacity of systems to maintain essential functions when exposed to challenges is called phenotypic robustness. Robustness is central to evolvability, because it allows an evolving population to explore new genotypes without detrimentally affecting essential phenotypes. Related terms are developmental stability, the ability to produce a robust phenotype when faced with challenges during development; and canalization, when genetic systems under long-term stabilizing selection evolve to a state of increased stability (157, 258–260). To that end, organisms have evolved systems to buffer the impact of mutations, noise and untoward environmental events.

The trait of robustness is pervasive in biology at every organizational level including protein folding, gene expression, and the neural connectome, as well as in physiological homeostasis, development, organism survival, species persistence, and ecological resilience (256, 261–265). In successful organisms, however, the trait of robustness exists in balance with trait evolvability. It seems a paradox; a robust system is resistant to generating new phenotypes while an evolvable system takes advantage of mutations to generate phenotypic variations (266). However, a robust system is only static with respect to essential morphological characteristics, that are said to be “deeply canalized.” If perturbations always led back to the native state, organisms would find it difficult to contend to unfamiliar challenges (262). A better evolutionary strategy is to protect essential native functions in the face of unexpected tumult while evolving new ones to adjust to challenges. This kind of robustness is not a barrier to evolution, but enhances it. It enables genotypic variability and new phenotypes without untoward functional consequences (24, 256, 267).

### Robust but Fragile

The mechanisms that successful organisms employ to ensure robustness are sufficiently flexible to support change when circumstances demand. Yet the balance between robustness and evolvability is tenuous and contains the germs of fragility. Robustness supports evolvability but the genome and the neural connectome are vulnerable to devastating disturbances. It is the balance between robustness and evolvability that may be relevant to complex diseases and mental disorders. The balance is maintained by two kinds of buffering systems, one structural, the other dynamic.

Modularity, for example, is a way most complex systems are structured. Modularity promotes stability by containing perturbations, reducing the interdependence of events and minimizing system-wide impact. Modules optimize network function because they are energy-efficient (268). They also confer evolvability by reducing constraints on change (269). Modular systems, however, are vulnerable to unexpected perturbations. This “robust yet fragile” trade-off is fundamental to complex dynamic systems (262, 270). Other mechanisms that are at once stabilizing and supportive of evolvability, such as redundancy, gene duplication, feedback control, and bow-tie architecture, are also vulnerable to catastrophic failure in the face of unusual stressors (267, 269). Gene duplication, for example, is a way to lower the intrinsic noise in gene expression. Increased copy number provides stability by preserving the native function of the gene when copies happen to mutate (206). We have already met some of the catastrophic failures that cometimes accompany copy number variation.

When new genes arise, systems exist to suppress their expression. Novel genes are not necessarily eliminated but survive from one generation to the next. The result is a vast pool for potential change, known as cryptic genetic variation (CGV). It is invisible under normal conditions but when circumstances change it is fuel for evolution (271). The hidden genes are a massive cache of adaptive potential. On the other hand, they are also a reservoir of potentially deleterious alleles (272).

When a robust system is stressed beyond its level of tolerance, phenotypic expression may be decanalized, which leads to increased phenotypic variability (273). A population moving beyond its adaptive niche challenges its genome to respond by increasing trait variability; decanalization is thus an agent of evolvability. At an individual level, crossing the threshold of stability opens the opportunity for cryptic alleles to express themselves, sometimes in untoward ways. Decanalization has been proposed to explain missing heritability in complex disease (273–275).

In diverse model organisms, the threshold of robustness differs among individuals; those with decreased robustness show increased penetrance of mutations and express previously cryptic genetic variation. It is not unlikely that phenotypic robustness also differs among humans. Individuals with lower robustness are thus more responsive to genetic and environmental perturbations and more susceptible to disease (157, 275). This is particularly relevant to disorders like autism and schizophrenia, where environmental events such as intrauterine exposure or obstetrical suboptimality may evoke a latent genetic proclivity.

Brain may be particularly vulnerable to decanalization because its development and activity are instructed by more than half of the genome, each allele with a different level of robustness. The hominid neocortex has expanded considerably, compared with closely related species; perhaps there hasn't been sufficient time to evolve robust cortical developmental trajectories. Brain has tightly-regulated critical windows of development; thus, there are few opportunities to compensate for perturbations (274).

### Versatile Proteins

Heat-shock proteins (HSP) are examples of a second type of buffering mechanism, dynamic and dependent on the expression of versatile proteins. HSP are protective against a wide range of environmental stresses, notably those known to be prenatal risk factors for neurological and psychiatric disorders, such as viral infection, hypoxia, inflammation, irradiation, alcohol, maternal seizure, and methylmercury (276–279). They are also genetic buffers, residing at the boundary between evolutionary stasis and change (264, 280).

HSP are molecular chaperones that play an essential role by protecting their “client” proteins from misfolding, for example, in the face of heat stress (281, 282). Like many genes and proteins, they are so-named after their earliest reported role; HSP are induced by high temperatures. They are an essential element of the biological stress response (281, 283). All species, from prokaryotes on up, have HSP genes; HSP expression is correlated with resistance to stress; and species' thresholds for HSP expression are correlated with the levels of stress that they naturally undergo (284). One HSP, hsp90, is necessary for the glucocorticoid receptor to develop (285) and regulates receptor activity (286). It participates in hormone signaling (280, 287) and restores hypothalamic–pituitary–adrenal homeostasis after stressful events (288). The aging-related decline in stress tolerance is associated with a lower capacity to generate stress proteins in general and HSP in particular (289, 290).

Genetic mutations are an important source of abnormal and misfolded proteins, and HSP buffer their effects as well (291). When an abnormal protein is expressed by a mutant gene, chaperones such as hsp90 participate in its degradation. Thus, HSP can buffer (i.e., suppress) phenotypic variation (283, 292–295). When genetic variations are “decoupled” from phenotypic expression, however, cryptic mutations accumulate (262, 293, 294, 296, 297). In light of this action, demonstrated in studies of fruit flies, zebrafish, bacteria, yeast, fungi and plants, hsp90 is called a “genetic capacitor” (282, 292, 294, 298, 299). It buffers against mutations and thus contribute to the robustness of the phenotype. When buffering operates normally, it prevents the development of abnormal phenotypes; if the potential phenotype were autism or schizophrenia, an effective buffering system might prevent the condition even in individuals with genetic or environmental risk factors (299, 300). Buffering capacity is finite, however, in some individuals more so than others. When an individual's buffering capacity is exceeded by excessive or unusual perturbations the result is increased phenotypic variation (292, 301, 302). In populations, this stress-sensitive storage and release of suppressed alleles may favor adaptive evolution (299, 303). In individuals, the consequences may not be quite so sanguine.

Fetal development has to be insulated from the damaging impacts of environmental and genetic perturbations to produce highly predictable phenotypes (300). The relevance of HSP to NDD is highlighted by their known effects on development; hsp90, for example, occupies a critical position in development because most of its client proteins are signal transducers (282, 292, 304–306). Because cell division is such an active process during fetal life, the opportunities for genetic mistakes to occur—especially in developing neurons—are legion. From studies in various organisms we have learned that many HSP buffer developmental perturbations on morphological traits (300, 305). In embryonic mice, exposure to subthreshold levels of environmental toxins induces HSP activity; inhibition of HSP leads to structural brain abnormalities and epileptogenesis (307).

In experimental animals, HSP activity can be inhibited in various ways. In real life, HSP is naturally variable and individuals differ in their capacity to generate buffering activity. There is substantial inter-individual variation in HSP induction during embryonic development of the central nervous system, where chaperones are essential to neuronal differentiation and survival (308). Embryos with stronger induction of HSP are less likely to be affected by inherited mutations; their development is more robust because mutations are less likely to be expressed (309). Clinically, Individual differences in HSP buffering are also associated with vulnerability to heart disease (310, 311) and the likelihood of extreme longevity (312, 313).

Throughout life, HSP concentrations are sensitive to tissue damage or destruction; concentrations are higher in patients with cardiovascular and autoimmune disease (314). HSP are induced in response to brain pathology; e.g., stroke, neurodegenerative disease, epilepsy, and trauma. One in particular, hsp90, is constitutively expressed in brain throughout life (315) and is especially abundant in limbic system-related structures such as the hippocampus (316). It is necessary for efficient neurotransmitter release at the presynaptic terminal and the development of receptors in the post-synaptic membrane (317).

The decline of HSP response with aging may be a cause of neurodegenerative disease (318). If unfolded or misfolded proteins are not recognized by HSP, they are capable of forming aggregates (319). Conversely, a vigorous HSP response reduces amyloid production (320, 321) and inhibits the aggregation of tau protein (322), alpha-synuclein (323), and huntingtin (324, 325). It induces the clearance of aggregates by autophagy (323, 326, 327).

Studies of HSP in autism and schizophrenia show higher levels of hsp70 (279, 328, 329) and increased auto-antibodies to hsp60, 70, and 90 and CRP40, a catecholamine-regulated heat-shock-like protein (33–278, 278–328, 328–334). In neural stem cells from schizophrenic patients, there is higher variability in the levels of HSF1, an HSP transcription factor (307). Interestingly, HSP levels are elevated in patients with temporal lobe epilepsy and lupus who are also psychotic, but not in those who are not (287, 307, 335).

The cited studies are by no means definitive. They are compromised by inconsistent findings and results that may be affected by the medications patients are taking. Nevertheless, it seems to be a promising area to pursue. Perhaps there is an intrinsic or acquired weakness in normal neuroprotective mechanisms in autism and schizophrenia (307, 328). Perhaps, too, the proper approach to disorders arising from the interactions of so many genes and genomic variants might dwell in the “hubs” (291, 295) and “bow-ties” (336, 337) that reside along the trajectory from genotype to phenotype, and represented by the heat-shock proteins (327).

### Variable Buffering

The approach may be promising but it won't be easy. HSP are an extended family of more than 100 proteins, traditionally identified by their molecular weight (from 8 to 110 kDa). Each category includes multiple proteins, many of which have multiple isoforms, not all of which are easily measurable (338–340). Individual HSP vary considerably in their expression, protein structure, localization, and ability to be induced. Complicating matters, the buffering capacity of an individual HSP is guided by multiple “co-chaperones” (291).

The behavior of HSP is not consistent. Hsp90, for example, protects the cell from genetic variation; or it may have the opposite effect, rescuing proteins that arise from mutations with folding or stability defects (341). Thus, they may maintain mutated proteins in a partially active state, permitting them to persist within the cell, to aggregate or cause some other mischief (327).

Buffering mechanisms also operate at different levels among the multiple components that contribute to every polygenic trait (342). The subunits that participate in a complex trait—single genes, or sets of well-integrated genes—may be robust or not. Subunits most likely to be robust are ones with high mutation rates, often at the expense of reduced robustness of genes or subunits with lower mutation rates. This may pose constraints or lead to conflicts that influence the buffering of the unit as a whole (256).

Nor do HSP operate in a vacuum. Whether a chaperone acts as a buffer, lessening mutational effects, or as a potentiator, increasing mutational effects, is not a fixed property of the protein itself but is influenced by the different mutations with which it interacts (343). The actions of HSP are also influenced by their genetic background (157, 344, 345). We don't know much about the genes that participate in the heat-shock response in humans, but 59 genes (7 positive activators and 52 negative regulators) participate in the heat-shock response in *Caenorhabditis elegans* (346). In *Saccharomyces cerevisiae*, no fewer than a thousand genes have been identified that alter sensitivity to heat shock (347).

The thresholds of robustness mechanisms are not only *a priori* variable, but are influenced by epigenetic changes that occur throughout one's life and that occurred in generations past (172, 283, 293, 301, 309, 348–350). Genetic variation present in one generation can influence phenotypic traits in the next, even if individuals do not inherit the variation. The environment experienced by one generation can influence phenotypic variation in the next several (351).

The critical balance between stability and variability, therefore, hovers on the fine edge of criticality. The cell has developed an exquisite system to prevent or mitigate destabilizing events and thus preserve its integrity and that of the organism. Understanding and possibly manipulating the agents that buffer cells from destabilizing agents holds at least some promise as a therapeutic approach to complex phenotypes like cancer and autoimmune disease (352–355). It may be a frail reed in the face of the complexity of genomic behavior but is one that is close to hand.

### If Things Were Simple, Word Would Have Gotten Around

Individual differences in buffering capacity and genetic background are two of many elements that contribute to the variable and unpredictable expression of clinical phenotypes. Is it possible, therefore, to make useful predictions about the phenotypes of individuals from their complete genome sequences? The “typical” phenotypic outcome of an individual's genome may well be predictable, but it is much more difficult to predict the actual outcome for a particular individual. Individual outcomes are no more than probabilistic. The genome, like the neural connectome, exists in a metastable state, a critical, narrow edge between order and chaos. In such states, irreducible uncertainties and unexpected hazards are always present. The premises of “personalized medicine” look more like Laplace's demon every day.

The problem, for a personalized medicine demon, is that the relation of genotype and the phenotype of complex traits is decidedly non-linear. Non-linearity is characterized by sudden changes in phenotype with small changes in genotype; thus, not all genes are equally correlated with the trait whose ontogeny they control (356). Non-linearities are a ubiquitous feature of development and gene expression networks (357–364). Non-linearities are given to sudden discontinuities and can lapse, unexpectedly, into catastrophe (365–367).

Non-linearity is a bother to demons but is necessary for robustness to co-exist with evolvability. In model genotypes with low levels of non-linearity, stability is the rule; those with non-linear dynamics allow expression levels to be robust to small perturbations, while generating high diversity under larger perturbations; that is, evolvability (368, 369).

Genetic variation influences the phenotype by processes that act at different scales, times, and locations within the organism (182, 364, 370). The trajectory from genotype to phenotype, therefore, is not only complicated, it is complex. At every step, one is confronted with complex systems composed of parts that are complex systems in their own right and complex systems tend to show surprising and unexpected behavior. The behavior of every system is affected by interactions, direct and indirect, with all the others. The individual and his destiny can't be understood in terms of one or two such sub-systems, or even all of them together.

The individual components of complex systems interact in manifold ways, including highly dynamic regulatory and feedback mechanisms (371). Within this framework, a single cause can produce multiple and unpredictable effects and even small fluctuations can have unexpected consequences. Linear casual explanations—that conceive reality as a linear succession of elementary events from cause to effect—are usually unable to describe how complex systems behave (372, 373).

One struggles, therefore, to generate clinical insights from the information we have gleaned from years of study of our two most important adaptive systems:

- Complex systems may never be given to complete descriptions, or unchanging and non-provisional rules to control their behavior (374).
- We still know frustratingly little about how changes in genotype determine the changes in phenotype (341).
- The “sequence space” of the genome is so vast that an exhaustive functional mapping and characterization of epistasis for any protein or gene is nigh-on impossible (375).
- The genetic information we are lacking about traits and diseases is potentially immense (376).
- The phenotype of each individual is usually considered as an interaction between two variables: the genes each individual carries and the environment that they experience… (but) the evidence suggests this is not always the case (351).
- It is futile to seek the basis of autism and schizophrenia amidst the profound complexity and variability of genetic and neural networks. Our present nosological constructs are imperfect, only “umbrella terms” that comprise a heterogenous mix of “real” conditions (377, 378).
- The complexity of brain function and structure is not reflected in current psychiatric disease nosology (238).

If such were really the case, we would be left with nothing but truisms: complex systems like the genome and neural connectome are “intrinsically and irreducibly hazardous” (45). Or rhetorical flourishes: “The same “genes” that drive us mad have made us human” (379). I prefer to think that our “umbrella terms,” fuzzy sets as they are, may be the best way to translate the complexities of genomics and connectomics in a meaningful way. Mental disorders, I think, are as variable and mutable as the neural networks and the genome whence they arise. They are as variable and unpredictable as people are.

I prefer to leave the reader with more than an intellectual dead-end. Appreciating the complexity of the genome is an opportunity to revise our expectations of how it generates complex disease and how we may address the crucial issue of prevention.

About genes:

- The genome is not a static blueprint but a dynamic participant in the affairs of the cell.
- There is more to genomic exploration than SNPs and QTLs.
- What matters about the genome is not only its base-pair sequence but its behavior, especially its interactions with the environment, which is mediated by proteins and RNAs of various stripe.
- Genomic expression is intrinsically noisy, stochastic, and unpredictable.
- The dynamic and mutable nature of gene expression may be a source of species adaptability but it is also a potential source of individual vulnerability.

About mutations:

- They occur frequently, especially in the human genome.
- Mutations occur more often in specific regions of the genome and are more likely to occur in some individuals.
- Mutations that are only slightly deleterious (or beneficial) are subject to weak selection (380).
- Therefore, mutations accumulate, a reservoir of potential adaptions for the species but, on occasion, of disastrous events in the lives of individuals (381).

About phenotypes:

- From transcription to RNA processing, translation, and protein folding and all the way up to protein activity and cellular fitness, there are many layers of biological organization where the effects of mutation can be transformed (382).
- To make predictions about the phenotypes of individuals, it is clear that knowledge of genome sequencing is usually insufficient. Rather, we need to consider how genetic, environmental and stochastic variation, together with transgenerational effects, combine to determine the phenotypes of individuals (351).
- The processes that govern the trajectory from phenotype to genotype hover on a critical edge between stability and variability.
- The spectrum of outcomes can be related to continuous functions in variable elements.
- Catastrophic outcomes may be related to non-linear functions.

Complex traits, including complex diseases, are attributable to “multiple genes of small effect”--among which are included all the sources of genomic, proteomic and neuronal variability. Nevertheless, complex traits exist and although quite variable, they occur with sufficient regularity to allow reliable descriptions and exhibit behavior that is more-or-less consistent. It is this, rather than their variability, that should entertain our interest. A current explanation is that mutations occurring in many different forms and at hundreds of targets converge on a much smaller number of molecular, cellular and anatomical pathways critical to the development and functioning of the CNS (383–385). The assumption is that aberrations in the expression of a great many genes affect a much smaller number of developmental pathways. It reflects a characteristic of complex systems: hierarchical networks are more concentrated higher up the scale. Although we suspect that developmental pathways are fewer in number than the genes involved, this is merely an assumption (360). However, we know that gene expression is processed through a much smaller number of “bow tie” processes that govern the generation of phenotypes and that maintain robustness without compromising evolvability. They deserve critical analysis.

At the level of pure theory, we may entertain a speculation about how primate evolution and then evolution of the hominids was so successful in the face of such a high degree of variability—to a point where even mechanisms designed to buffer it from perturbations are as variable and unpredictable. Ironically, an answer may be gleaned from computer simulations and studies of evolution among cancer cells and bacteria. From such studies, we learn that reproductive fitness is not the only goal of natural selection, nor is adaptation. Populations with lower initial fitness systematically adapt more rapidly than populations with higher initial fitness. Genotypes with lower fitness are more adaptable, over the long run, than those with higher fitness (381, 386–388).

“It is tempting to suggest that the promiscuity inherent in biology is tolerated with minimal detriment rather than corrected at high cost” (389). The promiscuity inherent in biology is not only tolerated, it is put to good use. If anything, it prevents a species from achieving a “genetic optimum.” The hydra have achieved a genetic optimum, I suppose, and so have crocodilians and turtles. But their optimum is really only a genetic ceiling. Hominids have achieved adaptability, not a genetic optimum. As a species, we are fit but not perfect. Adaptability is not a perfect fit to one's niche but the capacity to accommodate a wide range of potential environments. It is the genomic flexibility that allows small and large changes to be made in response to change. To that end we have evolved the trait of evolvability, which is manifest in variability at every point on the genotype-to-phenotype map. Trait evolvability, however, exists in balance with trait robustness. The balance is not perfectly balanced, however. It is distributed unevenly. It confers stability to most of us, albeit in graded fashion; less to many and very little to an unfortunate few.

## Author's Note

My hypothesis is that evolvability is relevant to the problems of psychiatric genetics: the problem of missing heritability, for example; of the variable expression of neurodevelopmental disorders arising from a particular genetic aberration; and the intriguing problem of autism and schizophrenia, disorders that are highly heritable but persist in spite of conferring low reproductive success. Genomic variability drove the evolution of neural complexity; it was also fuel for the evolvability of the hominid lineage. The paper is a review of mechanisms of genomic variability that are well-developed, scientifically, and also clinically relevant. The genetic elements that conferred variability and evolvability happen to be over-represented in autism and schizophrenia. The relevance of evolvability to neuropsychiatric disorders is illustrated by the robustness of the human genome, its ability to maintain stability in the face of genomic variability; yet it is also uniquely evolvable. The balance between robustness and evolvability is illustrated by buffering mechanisms that are structural or dynamic. Both, however, are fragile and subject to a high degree of inter-individual variation. The paper introduces a novel way to think about the genetics of neuropsychiatric disorders in particular, complex traits and complex disease in general.

## Author Contributions

The author confirms being the sole contributor of this work and has approved it for publication.

## Conflict of Interest

The author declares that the research was conducted in the absence of any commercial or financial relationships that could be construed as a potential conflict of interest.

## Footnotes

^1^Joseph Heller, Catch-22.

^2^Sontag S. Illness as metaphor and AIDS and its metaphors. Toronto: Doubleday; 1990, page 36.

^3^Mutant clones are often the source of treatment resistance. Mutant clones that are resistant to chemotherapy may persist in a dormant state, only to erupt at a later time.
